# Susceptibility and Immune Defence Mechanisms of *Rhynchophorus ferrugineus* (Olivier) (Coleoptera: Curculionidae) against Entomopathogenic Fungal Infections

**DOI:** 10.3390/ijms17091518

**Published:** 2016-09-09

**Authors:** Abid Hussain, Muhammad Rizwan-ul-Haq, Hassan Al-Ayedh, Ahmed Mohammed AlJabr

**Affiliations:** 1 Laboratory of Bio-control and Molecular Biology, Department of Arid Land Agriculture, College of Agricultural and Food Sciences, King Faisal University, Hofuf 31982, Saudi Arabia; abhussain@kfu.edu.sa (A.H.); malhaq@kfu.edu.sa (M.R.-u.-H.); 2 Life science and Environment Research Institute, King Abdulaziz City for Science and Technology, P.O. Box 6086, Riyadh 11442, Saudi Arabia; alayedh@kacst.edu.sa

**Keywords:** immune defence, feeding performance, immune-related genes, red palm weevil, virulence

## Abstract

Insects infected with entomopathogenic fungi, experience physiological changes that influence their growth and immune defence. The potential of nine isolates of entomopathogenic fungi was evaluated after determining percent germination and relative conidial hydrophobicity. However, nutritional indices were evaluated after immersing eighth-instar *Rhynchophorus ferrugineus* larvae into each isolate suspension (1 × 10^7^ conidia/mL). The results showed that isolates B6884 and M9374 had 44.51% and 39.02% higher conidial hydrophobicity compared with isolate I03011 (least virulent). The results of nutritional index assays revealed a significant reduction in growth indices after infection with different isolates. Compared with control, B6884 and M9374 greatly decreased larval growth by reducing the efficacy of conversion of ingested food (36%–47%) and Efficacy of conversion of digested food (50%–63%). Furthermore, only isolate B6884 induced 100% mortality within 12 days. Compared with control, isolate I03011, possessing the lowest conidial hydrophobicity, only reduced 0.29% of the efficacy of conversion of ingested food (ECI) and 0.48% of the efficacy of conversion of digested food (ECD). Similarly, transcriptomic analysis of genes related to the Red palm weevil (RPW) immune response, including pathogen recognition receptors (*C-type lectin* and *endo-beta-1,4-glucanse*), signal modulator (*Serine protease-like protein*), signal transductors (*Calmodulin-like protein* and *EF-hand domain containing protein*) and effectors (*C-type* lysozyme, *Cathepsin* L., *Defensin-like protein*, *Serine carboxypeptidase*, and *Thaumatin-like protein*), was significantly increased in larval samples infected with B6884 and M9374. These results suggest that for an isolate to be virulent, conidial hydrophobicity and germination should also be considered during pathogen selection, as these factors could significantly impact host growth and immune defence mechanisms.

## 1. Introduction

Red palm weevil (RPW) is an exotic highly destructive pest of palms, particularly *Phoenix dactylifera*, in different geographical areas worldwide. The legless, creamy white larvae (grubs) of *Rhynchophorus ferrugineus* (Olivier) (Coleoptera: Curculionidae) are the most destructive stage of the weevil. These insects feed on tender soft palm tissues and move toward the centre of the infested host palm. Under severe attack, such a feeding pattern causes the crown to collapse [1,2].

The control of the RPW primarily relies on the frequent use of synthetic pesticides [1]. These practices are not sustainable, endanger biological diversity, and deteriorate environmental quality. These setbacks have led to the search for alternative methods of RPW control, particularly bio-control agents. Previous studies have reported the use of bacteria [3], fungi [4,5,6], nematodes [7], and parasitoids [8] against RPW. However, the use of entomopathogenic fungi, is a promising alternative for reversing the hazardous dependence of agriculture on synthetic insecticides. The initial step toward the development of mycoinsecticides should involve laboratory evaluation to grade the tested isolates in terms of virulence [9].

The virulence of entomopathogenic fungal isolates primarily depends on a series of complex factors, including conidial hydrophobicity, germination, polarity, and fungal hydrolytic enzyme activities. The conidial hydrophobicity of entomopathogenic fungal isolates varies with the developmental stage of the fungus. Previous studies have characterized the cysteine-rich hydrophobin genes from *Metarhizium anisopliae* and *Beauveria bassiana*. Furthermore, these findings provided evidence of the involvement of these genes in the adhesion and virulence of entomopathogenic fungal isolates [10].

Host immune defence mechanisms are triggered immediately after entomopathogenic fungal spores invade and compromise the integrity of the host cuticle. Subsequently, a struggle between the host and pathogen is initiated, potentially leading to infection (compatible interaction) or disease resistance (non-compatible interaction). This host immune defence primarily involves different innate immune reactions, such as cellular and humoral immune responses [11]. Thus, the host cuticle covering the entire body provides the first line of immune defence against invading pathogens. The pathogens breach the cuticle through the production of proteases [12,13]. In addition, insects display cellular immune responses, such as self-defence. However, the invading pathogens release toxins (e.g., destruxins) to reduce the impact of host immune responses. Once the pathogen has overcome the physical barriers, the host membranes produce molecular structures that secrete proteins involved in host defence to cope with the pathogen attack [14]. The fat body is a major immune responsive organ involved in the synthesis of unique antimicrobial peptides (AMPs) targeting specific microorganisms. There have been a few scattered reports on the immune responses of red palm weevils induced through nematodes [15] and bacteria [16,17,18]. Currently, there are no studies concerning the molecular mechanisms of the RPW immune response induced through entomopathogenic fungi. Thus, the present study was designed to evaluate the potential of entomopathogenic fungal isolates, determine the effect of the tested fungal isolates on the growth and development of *R. ferrugineus* larvae, and gain insights into the molecular mechanisms of the red palm weevil immune response against invading entomopathogenic fungal microbes to improve *R. ferrugineus* control strategies.

## 2. Results

### 2.1. Conidial Virulence-Related Traits Evaluation of the Entomopathogenic Fungal Isolates

The relative conidial hydrophobicity significantly varied (*F* = 22.6; df = 8, 36; *p* < 0.0001) among the tested isolates. Isolate B6884 showed the highest relative conidial hydrophobicity, and I03011 showed the lowest conidial hydrophobicity. The lethal concentration to kill 50% populations (LC_50_) significantly varied (*F* = 14.0; df = 8, 36; *p* < 0.0001). The least virulent isolate of *Isaria fumosorosea* (I03011) showed the highest LC_50_ value at 3.29 × 10^9^ spores/mL. However, the highly virulent *B. bassiana* (B6884) isolate showed the lowest median lethal concentration at 4.59 × 10^7^ spores/mL against 8th-instar red palm weevil larvae (Table 1). However, non-significant differences in conidial germination were observed among all the isolates, except I7284, B3H203, and I03011 (*F* = 97.3; df = 8, 36; *p* < 0.0001). All isolates showed ≥96% viability, except isolates B3H203, I7284, and I03011 (Table 1). Results obtained from the virulence-determining traits evaluated in the current study enable us to suggest that germination percentage and relative conidial hydrophobicity significantly affect the virulence of the tested isolates.

### 2.2. Mortality of R. ferrugineus

**Figure 1:**
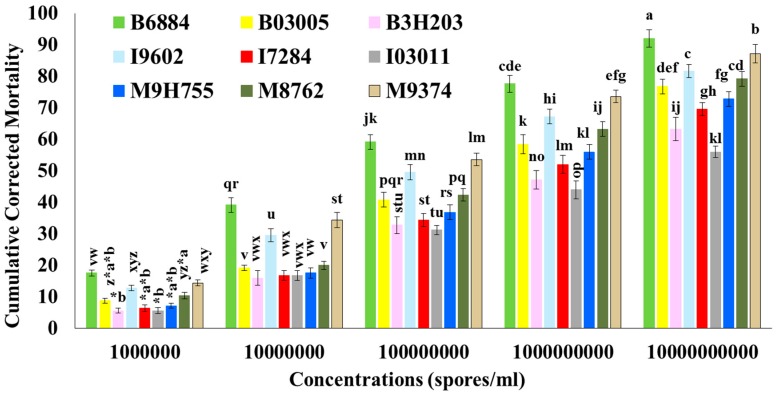
Cumulative corrected percent mortality of *Rhynchophorus ferrugineus* larvae infected with different concentrations of the tested isolates of entomopathogenic fungi. Means ± SE values followed by different letter(s) are significantly different. (Fisher’s least significant difference (LSD) test, α = 0.05).

The concentration mortality response of 8th-instar red palm weevil larvae against the tested entomopathogenic fungal isolates was significantly different among fungal isolates (*F* = 178.44; df = 8, 160; *p* < 0.0001), concentrations (*F* = 326.11; df = 4, 160; *p* < 0.0001), and interactions (*F* = 2.23; df = 32, 160; *p* < 0.0001). Overall, the concentration-dependent mortality response of red palm weevil larvae was observed among all tested isolates. Conidial suspensions at higher concentrations induced higher mortality (Figure 1).

The virulence of all studied isolates against red palm weevil larvae at a concentration of 1 × 10^7^ conidia/mL significantly differed at different time intervals (*F* = 768.49; df = 2, 96; *p* < 0.0001), isolates (*F* = 247.28; df = 8, 96; *p* < 0.0001), and their interactions (*F* = 6.14; df = 16, 96; *p* < 0.0001). The conidia of isolate B6884 were more pathogenic, showing the lowest lethal time to kill 50% (LT_50_) (3.96 days) (Table 2). Only the isolate B6884 caused 100% mortality within 12 days of experimentation. However, isolates M9H755, I7284, B3H203, and I03011 caused the lowest mortality, at 56.00%, 52.80%, 51.20% and 50.40%, respectively, even after 12 days post-infection (Table 2). The LT_50_ values also significantly varied (*F* = 76.8; df = 8, 36; *p* < 0.0001). The highest LT_50_ value, calculated from the infection of isolate I03011 (11.51 days), remained significantly at same level with isolates I7284 (11.21 days) and B3H203 (11.37 days).

### 2.3. Quantification of Host Immune Defence-Related Genes Using Quantitative Reverse Transcription Polymerase Chain Reaction (qRT-PCR)

**Figure 2:**
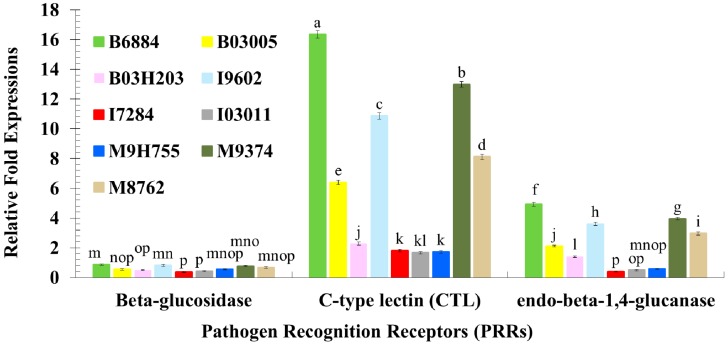
Relative fold-expression of pathogen recognition receptors (PRRs) of *Rhynchophorus ferrugineus* larvae in response to fungal infections using quantitative real-time PCR. Means ± SE values followed by different letter(s) are significantly different. (Fisher’s Least Significant Difference (LSD) test, α *=* 0.05).

Highly-virulent isolates of entomopathogenic fungi, such as B6884 and M9374, established in the current study greatly induced the expression of immune related genes of red palm weevil larvae. The interaction of the quantitative expression of pathogen recognition receptors (PRRs) from the fat body of red palm weevil larvae with tested fungal isolates was significantly different (*F* = 357.14; df = 16, 108; *p* < 0.0001). Overall, *C-type lectin* (CTL) was significantly expressed compared with other studied PRRs (Figure 2). However, *Beta-glucosidase* was not greatly expressed, showing <1% relative fold-expression compared with control. Overall, isolates B6884 and M9374 significantly induced the expression of *C-type lectin* and *endo-beta-1,4-glucanse* of red palm weevil larvae (Figure 2). Isolates I7284, I03011, and M9H755 had the lowest effect on the expression of *C-type lectin* and *endo-beta-1,4-glucanse* genes.

**Figure 3:**
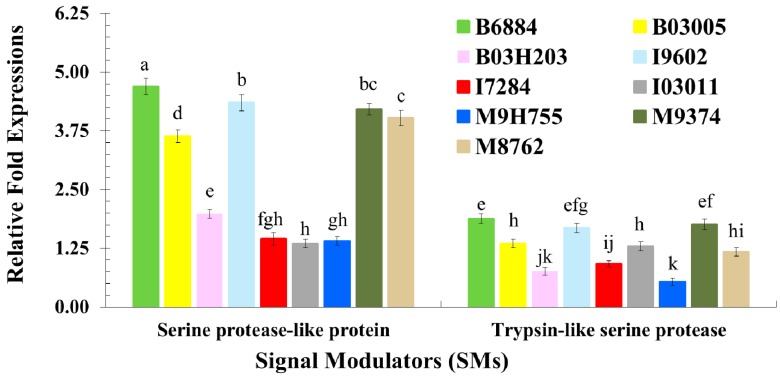
Relative fold-expression of signal modulators of *Rhynchophorus ferrugineus* larvae in response to fungal infections using quantitative real-time PCR. Means ± SE values followed by different letter(s) are significantly different. (Fisher’s Least Significant Difference (LSD) test, α *=* 0.05).

Quantitative expression pattern of signal modulators, such as *serine protease-like protein* and *trypsin-like serine protease*, among the tested larvae in response to infections, showed great variations (Figure 3) The gene expression of the studied signal modulators (*F* = 1071.42; df = 1, 72; *p* < 0.0001), upon exposure to different fungal isolates (*F* = 129.87; df = 8, 72; *p* < 0.0001) and their interactions (*F* = 45.73; df = 8, 72; *p* < 0.0001), showed significant differences. Comparatively, *serine protease-like protein* was significantly expressed compared with *trypsin-like serine protease* (Figure 3). Overall, the most virulent isolate, B6884, induced the highest expression of *serine protease-like protein*. The lowest fold-expression of *serine protease-like protein* and *trypsin-like serine protease* was induced by the least virulent isolates, including I7284, I03011, and M9H755 (Figure 3).

**Figure 4:**
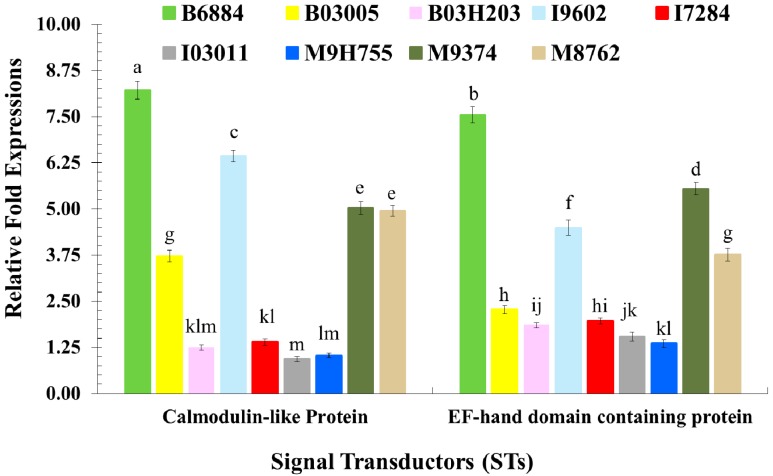
Relative fold-expression of signal transductors of *Rhynchophorus ferrugineus* larvae in response to fungal infections using quantitative real-time PCR. Means ± SE values followed by different letter(s) are significantly different. (Fisher’s Least Significant Difference (LSD) test, α *=* 0.05).

The expression of signal transductors, such as *Calmodulin-like protein* and *EF-hand domain containing protein* (*F* = 17.33; df = 1, 72; *p* < 0.0001), upon exposure with different fungal isolates (*F* = 513.64; df = 8, 72; *p* < 0.0001) and their interactions (*F* = 24.15; df = 8, 72; *p* < 0.0001), showed significant differences. Both signal transductors showed enhanced expression to the most virulent inoculum. For example, isolate B6884 significantly induced the expression of *Calmodulin-like protein* and *EF-hand domain containing protein*. However, I03011 and M9H755 did not induce the significant expression of the studied signal transductors, resulting in the lowest expression (Figure 4).

**Figure 5:**
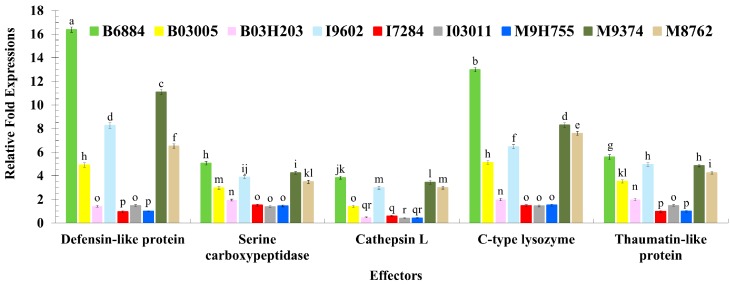
Relative fold-expression of effectors of *Rhynchophorus ferrugineus* larvae in response to fungal infections using quantitative real-time PCR. Means ± SE values followed by different letter(s) are significantly different. (Fisher’s Least Significant Difference (LSD) test, α *=* 0.05).

The up-regulation of the studied effectors, such as *C-type lysozyme*, *Cathepsin* L., *Defensin-like protein*, *Serine carboxypeptidase*, and *Thaumatin-like protein* (*F* = 1161.49; df = 4, 180; *p* < 0.0001), among the larvae infected with different fungal isolates (*F* = 2246.79; df = 8, 180; *p* < 0.0001) and their interactions (*F* = 101.06; df = 32, 180; *p* < 0.0001), significantly differed at twenty-four hours post exposure. Overall, the infection of isolates B6884 and M9374 significantly enhanced the expression of all studied effectors, particularly *Defensin-like protein*, *C-type lysozyme*, and *Thaumatin-like protein* (Figure 5). The isolates I7284, M9H755, and I03011 did not induce the expression of the studied effectors.

### 2.4. Impact of Entomopathogenic Fungi on the Growth of R. ferrugineus

The efficacy of conversion of ingested food (ECI) of the larvae upon exposure to all tested isolates of entomopathogenic fungi significantly differed (*F* = 5.22; df = 9, 39; *p* < 0.0001). Overall, larvae infected with B6884 exhibited the maximum ECI reduction (46.41%) compared with control larvae. A relatively negligible reduction (0.29%) in *R. ferrugineus* larvae infected with isolate I03011 was observed (Table 3). There were also significant differences in the efficacy of conversion of digested food (ECD) of larvae upon exposure to the tested entomopathogenic fungal isolates (*F* = 27.48; df = 9, 39; *p* < 0.0001). Isolate B6884 showed significantly declined ECD, resulting in a tremendous reduction (62.46%) in ECD compared with the control. However, two isolates of *I. fumosorosea*, I7284 and I03011, and one *B. bassiana* isolate B3H203 impart a <10% reduction in ECD compared with the control (Table 3). The approximate digestibility (AD), however, significantly increased upon exposure to different isolates (*F* = 10.95; df = 9, 39; *p* < 0.0001). The most virulent isolates B6884 and M9374 significantly enhanced the AD of the infected larvae, increasing the AD 42.78% and 29.49% compared with control larvae, respectively. However, the least virulent isolate I03011, I7284, and B3H203 only increased the AD 0.20%, 2.22%, and 1.80% compared with control larvae, respectively (Table 3). The reduction in ECI and ECD index directly proportional to the virulence of the isolates. Overall, virulent isolates, such as B6884 and M9374, impart the maximum reduction in the ECI and ECD indexes, whereas the least virulent isolates failed to inhibit growth indices of red palm weevil larvae. In contrary, inversely proportional relationship was observed between AD and virulence of the isolates compared to the ECI and ECD indexes (Table 3).

## 3. Discussion

The pathogenic and growth inhibition activities of naturally-occurring entomopathogenic fungi against insects of different orders have previously been reported [19]. Previous studies have given little consideration to the virulence-determining traits of entomopathogenic fungi and their impact on the growth and immune defence mechanism of an economically important pest of the date palm, *R. ferrugineus*. The present study showed that microbe virulence factors, including the germination percentage and relative conidial hydrophobicity, significantly affect the virulence of the studied isolates. Entomopathogenic fungal isolates with higher virulence, elicit higher expression of immune related genes, and slowing the larval growth, ultimately leading to higher mortality among infected larvae.

The current findings clearly indicated that conidial germination and hydrophobicity defined the virulence of the isolates. We found variations in germination and conidial hydrophobicity among the studied isolates. Conidial hydrophobicity is specifically provided through a group of small cysteine-rich proteins (hydrophobins), involved in conidial adhesion, virulence, and dispersion. Recently, an empirical evidence of the involvement of three hydrophobin genes *Hyd1*, *Hyd2* and *Hyd3* in virulence, hydrophobicity, and conidiation was provided [20]. In another study, the hydrophobin genes, *Hyd1* and *Hyd2*, were implicated in conidial hydrophobicity and, ultimately, the regulation of the level of adhesion and virulence of the strain [21,22]. Based on these findings, we suggested that pathogen virulence is important to invade the host. Conidia with high levels of hydrophobicity resulted in the high mortality of red palm weevil larvae. During fungal pathogen selection, virulence-determining traits, such as conidial hydrophobicity and conidial germination, reported in the current study should be thoroughly considered.

*R. ferrugineus* larvae infected with isolates B6884 and M9374 gained less weight compared with uninfected (control) larvae. Isolate B6884 remained the most virulent isolate, followed by isolates M9374 > I9602 > M8762 > B03005 > M9H755 > I7284 > B3H203 > I03011. Nutritional indices, particularly the ECI and ECD values of *R. ferrugineus* larvae, were significantly different, suggesting that virulent isolates tremendously reduced the growth of red palm weevil larvae. The ECI values representing the overall measurement of ingested food used by the larvae for growth were greatly reduced by the infection of isolate B6884 (46.41%), M9374 (36.59%), I9602 (29.25%), M8762 (20.00%), B03005 (14.88%), M9H755 (8.74%), I7284 (4.22%), B3H203 (2.67%), and I03011 (0.29%). The decrease in ECI values compared with the control revealed that most of the ingested food is used for energy to combat the invading pathogen, and less food is used for larval growth. These findings are consistent with a previous investigation in which *M. anisopliae* (strain 406) significantly reduced the ECI values against all of the studied larval instars of *Ocinara varians* Walker [23].

ECD, an index to calculate the precise insect biomass from digested food, was also decreased upon infection with the conidia of virulent isolates. This reduction might reflect the fact that digested food is metabolized for energy production, thereby reducing the ECD compared with the control [5]. In another study, similar growth inhibition pattern of ECI and ECD was reported in *O. varians* Walker larvae in studies of the virulence of entomopathogenic fungal strains [9].

The fungal infection of virulent isolates tremendously increased the AD values of the red palm weevil larvae. The enhanced AD values might reflect the fact that the fungus-inoculated nutrient-deficient larvae require energy for immune defence. This energy could only be obtained through the use of intrinsic abilities to enhance the approximate digestibilities of the limited foodstuff. Similar results were obtained when the pesticidal activity of labramin was evaluated against the Mediterranean flour moth [24].

It is clear from the mortality and nutritional indices results that isolates B6884 and M9374 are the most virulent. This virulence is mainly regulated by the conidial hydrophobicity that enhances the infection by facilitating conidial adhesion with the host cuticle. In case of compatible interaction (infection), the host immune defence mechanism is greatly up-regulated to combat infection and less energy remained for host growth. The host, under such circumstances (low energy), becomes weaker and more vulnerable to the sporulating conidia that starts their growth in vivo.

These findings also showed that isolates B6884 and M9374 enhanced the response of the pathogen recognition receptors, signal modulators, signal transductors and effectors responsible for host immune defence mechanisms. The enhanced activities of PRRs (particularly *C-type lectin* and *endo-beta-1,4-glucanse*); signal modulators (particularly *Serine protease-like protein*); signal transductors (*Calmodulin-like protein* and *EF-hand domain containing protein*); and effectors (*C-type* lysozyme, *Cathepsin* L., *Defensin-like protein, Serine carboxypeptidase*, and *Thaumatin-like protein*) reduced ECI and ECD values, revealing that the host spends most of its energy combating the invading pathogen attack; hence, little nutrition remains for growth and development. The strains with less conidial hydrophobicity and low germination remained less pathogenic and should not be incorporated into the integrated red palm weevil management programme.

## 4. Materials and Methods

### 4.1. Entomopathogenic Fungi

Nine isolates of three different genera of entomopathogenic fungi (*Beauveria bassiana*, *Isaria fumosorosea*, and *Metarhizium anisopliae*) were selected for experimentation (Table 4). The isolates were grown at 25 ± 0.5 °C and 70% ± 5% relative humidity on potato dextrose agar (Oxoid, Hampshire, UK) in complete darkness.

### 4.2. Rearing of Red Palm Weevils

RPW adults were collected from infested date palms. The weevils were reared on pineapples with a 16-h light photoperiod at 30 ± 1 °C and 75% ± 5% RH in cages (57.5 × 29 × 58 cm). After hatching, second instar red palm weevil larvae were individually shifted to 400-mL plastic cups (perforated) and provided artificial diets (Table 5) in an incubator under controlled conditions.

### 4.3. Virulence Evaluation of the Isolates of Entomopathogenic Fungi

#### 4.3.1. Conidial Germination

The conidial germination of the tested entomopathogenic fungal isolates was determined, as previously described [25]. Briefly, a 50-µL suspension of each studied isolate (24-day-old) at a conidial concentration of 1 × 10^7^ conidia/mL in 0.05% Tween 80 was used to inoculate Petri dishes (115 mm × 20 mm) containing PDA. The required concentration (1 × 10^7^ conidia/mL) for each fungal suspension was prepared using a Neubauer haemocytometer (Wertheim, Germany). The plates were incubated at 25 ± 0.5 °C, 70% ± 5% RH in complete darkness. Each Petri dish was regarded as one replicate. Five replicates were prepared. Percent conidial germination was determined after 18 h by counting total 100 conidia (germinated or ungerminated) from seven different fields of vision in single Petri dish under a compound microscope (×400). Conidium was considered “germinated” if the germ tube was more than half the diameter. The average of seven fields of vision from each plate was considered as one replicate. The data were analysed using one-way analysis of variance (ANOVA) and Fisher’s least significant difference (LSD) test for means comparisons [26].

#### 4.3.2. Conidial Hydrophobicity

An aqueous-solvent partitioning assay was used to determine the conidial surface hydrophobicity [27]. Briefly, the final concentration (1 × 10^7^ conidia/mL) of each tested isolate with five replicates was prepared using 0.1 M KNO_3_. The optical density (OD) of each experimental unit, referred to as the OD_total_, was determined at 660 nm using a spectrophotometer. Subsequently, a 6 mL suspension of each experimental unit was shifted to a universal bottle containing 2 mL of hexadecane. After agitation (20 s), the mixture was transferred into a separation funnel for aqueous phase separation. Subsequently, the optical density of the aqueous phase, referred to as the OD_aq_, was determined at 660 nm using a spectrophotometer. The relative conidial hydrophobicity of all the replicates (five) of each isolate was determined using the following equation:

Relative conidial hydrophobicity (%) = 100 {1 − (OD_aq_/OD_total_)}

#### 4.3.3. Mortality

Twenty-five newly-molted (8th instar) *R. ferrugineus* larvae were immersed for 10 s into the 30 mL suspension in glass beaker at concentrations of 1 × 10^6^, 1 × 10^7^, 1 × 10^8^, 1 × 10^9^, and 1 × 10^10^ conidia/mL of each studied isolate for use in concentration mortality bioassays under laminar air flow cabinet. Five replicates were prepared with each replicate comprising twenty-five larvae. Each replicate-inoculated larva was individually incubated in separate 400 mL plastic cups (perforated) containing artificial diets (Table 5) to minimize mortality as a result of the cannibalism observed in a preliminary study. The experimental units were incubated under a 16-h light photoperiod at 30 ± 1 °C and 75% ± 5% RH. The bioassays were repeated over time at different occasions.

The LT_50_ value of each tested isolate was determined using a single concentration of 1 × 10^7^ conidia/mL. Fungus inoculated insects and control (immersed into 0.04% Tween 80) larvae were fed in plastic cups (400 mL) containing diet (Table 5) under 16-h light photoperiod at 30 ± 1 °C and 75% ± 5% RH. Each larva was provided 1.5 g of artificial diet daily. However, the mortality was recorded until 15 days post-inoculation. Each experimental unit was assessed daily to record mortality and replace the old diet with a fresh diet. Five replicates were prepared with each replicate comprising twenty-five larvae. Each larva was individually incubated in separate 400-mL plastic cups (perforated) to minimize the chances of mortality as a result of the cannibalism observed in a preliminary study. The dead larvae were carefully transferred under a laminar airflow cabinet into a sterilized Petri dish containing a dampened filter paper. Outgrowths on the surface of the mycosed larvae were inoculated onto PDA and observed under a microscope to confirm the causal agent of larval mortality. The bioassays were repeated over time at different occasions. Control mortality was adjusted using Abbott’s formula [28]. The corrected percent mortality data were angularly transformed. In addition, the corrected angularly transformed cumulative percent mortality data were analysed using repeated measure ANOVA and Fisher’s LSD test for means comparison [29]. The lethal time to kill 50% (LT_50_) for red palm weevil larvae and the lethal concentration to kill 50% (LC_50_) for red palm weevil larvae was calculated using probit analysis.

### 4.4. Evaluation of the Host Immune Defence Mechanism against Entomopathogenic Fungi through the Quantification of Immune-Related Genes Using qRT-PCR

RPW larvae (8th instar) were infected with each isolate suspension (1 × 10^7^ conidia/mL by Tween 80, London, UK) after immersing five larvae for 10 s. Each larva was regarded as one replicate. Infected and control larvae were individually fed an artificial diet in an incubator in separate 400-mL perforated plastic cups under a 16-h light photoperiod at 30 ± 1 °C and 75% ± 5% RH. After 24 h, larvae were dissected in saline to extract total RNA from the fat body using a commercial kit (Cat # 73404; Qiagen, Hilden, Germany). Total RNA was reverse-transcribed using a commercially available kit (Cat # 6110; TaKaRa Clontech, Paris, France). All primers were designed from the gene sequences deposited in NCBI, as listed in Table 6. The quantification was performed, following the protocol of the SYBR^®^ Premix Ex Taq™ II (Clontech: RR820W) according to the manufacturer for the CFX96 Real-Time System (Bio-Rad, London, UK). The results of each experimental unit were compared with those of the control through the relative fold-expression obtained after transforming the obtained results into absolute values using the 2^−ΔΔ*C*t^ method [30]. The relative expression of each gene was set to 1 for the uninfected (control) treatment. Due to the stability of the *Beta actin*, it was used in the current study as house-keeping gene. Two factor completely randomized design and Fisher’s LSD test was used to analyse data in SAS (Dubai, UAE) [26].

### 4.5. Impact of Entomopathogenic Fungi on the Growth of R. ferrugineus

Eighth-instar (newly molted) *R. ferrugineus* larvae were infected with suspension (1 × 10^7^ conidia/mL in Tween 80) of each isolate after immersion for 10 s. The infected and control (immersed into 0.04% Tween 80) larvae were separately fed a measured artificial diet in plastic cups and incubated under a 16-h light photoperiod at 30 ± 1 °C and 75% ± 5% RH. After feeding on the diets for 72 h, the final larval weight, remaining diet and frass weight after drying were measured. These weights were used to calculate the AD efficacy of ECD, and efficacy of conversion of ECI on a dry matter basis [5]. Five replicates were prepared, with each replicate comprising 25 larvae incubated in separate perforated plastic cups. The feeding performance bioassays were also repeated over time. The data for the growth indices were analysed using ANCOVA with SPSS version 11.5 (SPSS Inc., Chicago, IL, USA, 2002). In all cases, the treatments (fungal isolate) were considered to be independent variables. The dependent variables and covariates were assigned according to Scott et al. [31]. In the case of AD, the food (artificial diet) consumed by the larvae was considered as the covariate, while the food (artificial diet) consumed minus frass produced by the larva was considered as the dependent variable. For ECI, the mass gained by the larva was considered as the dependent variable, and the food (artificial diet) consumed by the larva was considered as the covariate. For ECD, the mass gained by the larva was considered as the dependent variable, and the food (artificial diet) consumed minus frass produced by the larva was considered as the covariate.

## 5. Conclusions

In summary, these results revealed that isolates B6884 and M9374 are highly virulent and impart high mortality, disturb larval growth, and elicit high expression of immune-related genes. Differences in larval growth, immune response, and mortality among infected larvae might be greatly affected through relative conidial hydrophobicity and germination; however, other factors, such as toxin production, might also play an important role. Future research should focus on the host-pathogen genome interaction to fully explore this mechanism.

## Figures and Tables

**Table 1:** Pathogenicity-related traits of the isolates of entomopathogenic fungi.

Fungal Species	Percent Conidial Germination	Relative Conidial Hydrophobicity	LC_50_ (Spores/mL)
*B. bassiana* 6884	98.40 ± 0.51 ^a^	94.80 ± 1.88 ^a^	4.59 × 10^7^ ^f^
*B. bassiana* 03005	97.60 ± 0.51 ^a^	79.80 ± 1.77 ^c^	4.36 × 10^8^ ^cde^
*B. bassiana* 3H203	68.80 ± 1.93 ^b^	67.80 ± 2.08 ^ef^	1.85 × 10^9^ ^b^
*I. fumosorosea* 9602	98.20 ± 0.37 ^a^	88.80 ± 2.52 ^ab^	1.53 × 10^8^ ^ef^
*I. fumosorosea* 7284	69.20 ± 2.08 ^b^	72.20 ± 2.29 ^de^	9.88 × 10^8^ ^bc^
*I. fumosorosea* 03011	63.60 ± 3.09 ^c^	65.60 ± 2.87 ^f^	3.29 × 10^9^ ^a^
*M. anisopliae* 9H755	96.00 ± 1.14 ^a^	75.60 ± 2.04 ^cd^	7.22 × 10^8^ ^cd^
*M. anisopliae* 8762	97.20 ± 1.02 ^a^	86.40 ± 2.11 ^b^	3.08 × 10^8^ ^def^
*M. anisopliae* 9374	97.60 ± 0.93 ^a^	91.20 ± 2.29 ^ab^	8.20 × 10^7^ ^ef^

Means ± SE values within a column followed by different letter(s) are significantly different. The values are presented as the means of five replicates. (Fisher’s least significant difference (LSD) test, α *=* 0.05).

**Table 2:** Average corrected percent mortality of *Rhynchophorus ferrugineus* larvae infected with different isolates of entomopathogenic fungi.

Treatments	LT_50_ (Days)	Corrected Cumulative Percent Mortality at Different Time Intervals		
		4th Day	8th Day	12th Day
*B. bassiana* 6884	3.96 ± 0.20 ^e^	44.80 ± 2.33 ^de^	91.20 ± 2.33 ^a^	100.00 ± 0.00 ^a^
*B. bassiana* 03005	8.03 ± 0.31 ^c^	17.60 ± 1.60 ^h^	40.00 ± 2.83 ^e^	74.40 ± 3.25 ^b^
*B. bassiana* 3H203	11.37 ± 0.28 ^a^	6.40 ± 0.98 ^i^	19.20 ± 1.50 ^fgh^	51.20 ± 1.96 ^cd^
*I. fumosorosea* 9602	6.59 ± 0.28 ^d^	22.40 ± 2.04 ^fg^	49.60 ± 2.40 ^cd^	96.80 ± 1.50 ^a^
*I. fumosorosea* 7284	11.21 ± 0.40 ^a^	7.20 ± 0.80 ^i^	20.00 ± 1.79 ^fgh^	52.80 ± 2.33 ^c^
*I. fumosorosea* 03011	11.51 ± 0.38 ^a^	5.60 ± 0.98 ^i^	19.20 ± 2.33 ^fgh^	50.40 ± 2.04 ^cd^
*M. anisopliae* 9H755	10.14 ± 0.33 ^b^	6.40 ± 0.98 ^i^	23.20 ± 1.50 ^f^	56.00 ± 2.53 ^c^
*M. anisopliae* 8762	7.57 ± 0.24 ^c^	18.40 ± 1.60 ^gh^	41.60 ± 2.04 ^e^	76.80 ± 2.33 ^b^
*M. anisopliae* 9374	6.43 ± 0.27 ^d^	23.20 ± 1.50 ^f^	52.80 ± 3.20 ^c^	96.80 ± 1.50 ^a^

The numerical values represent the means of five replicates (*n* = 25). Means ± SE values followed by different letter(s) are significantly different. (Fisher’s Least Significant Difference (LSD) test, α *=* 0.05).

**Table 3:** Percent increase/decrease in nutritional indices of red palm weevils against different isolates of entomopathogenic fungi.

Treatments	Percent Inhibition Compared to Control		Percent Increase Compared to Control
	ECI	ECD	AD
*B. bassiana* 6884	46.41 ± 0.49 ^a^	62.46 ± 0.45 ^a^	42.78 ± 0.83 ^a^
*B. bassiana* 03005	14.88 ± 2.09 ^e^	21.85 ± 2.50 ^e^	9.02 ± 1.01 ^d^
*B. bassiana* 3H203	2.67 ± 0.74 ^g^	4.38 ± 1.01 ^gh^	1.80 ± 0.41 ^ef^
*I. fumosorosea* 9602	29.25 ± 0.99 ^c^	41.25 ± 0.79 ^c^	20.43 ± 0.67 ^c^
*I. fumosorosea* 7284	4.22 ± 0. 45 ^g^	6.48 ± 0.88 ^g^	2.22 ± 0.62 ^ef^
*I. fumosorosea* 03011	0.29 ± 0.03 ^g^	0.48 ± 0.05 ^h^	0.20 ± 0.02 ^f^
*M. anisopliae* 9H755	8.74 ± 0.60 ^f^	11.83 ± 1.25 ^f^	3.56 ± 0.94 ^e^
*M. anisopliae* 8762	20.00 ± 1.52 ^d^	33.07 ± 1.22 ^d^	19.54 ± 0.53 ^c^
*M. anisopliae* 9374	36.59 ± 2.99 ^b^	50.90 ± 2.80 ^b^	29.49 ± 1.57 ^b^

ECI (conversion of ingested food), ECD (conversion of digested food), and AD (approximate digestibility) represent the efficacy of conversion of ingested food, efficacy of conversion of digested food and approximate digestibility, respectively. The numerical values represent the means of five replicates (*n* = 25). Means ± SE values within a column followed by different letter(s) are significantly different. (Fisher’s Least Significant Difference (LSD) test, α *=* 0.05).

**Table 4:** Isolates of entomopathogenic fungi used in the experimentation.

Fungal Species	Isolate	Host	Origin	Date
*B. bassiana*	B6884	*Otiorhynchus ligustici*	Hungary	2001
*B. bassiana*	B03005	*Coptotermes formosanus*	China	2003
*B. bassiana*	B03H203	*Rhynchophorus ferrugineus*	Saudi Arabia	2012
*Isaria fumosorosea*	I9602	Coleoptera	China	2010
*Isaria fumosorosea*	I7284	*Hypothenemus hampei*	Mexico	2004
*Isaria fumosorosea*	I03011	*Coptotermes formosanus*	China	2003
*Metarhizium anisopliae*	M9374	*Listronotus maculicollis*	USA	2005
*Metarhizium anisopliae*	M9H755	*Rhynchophorus ferrugineus*	Saudi Arabia	2012
*Metarhizium anisopliae*	M8762	*Rhabdoscelus obscurus*	Australia	1996

**Table 5:** Nutritional ingredients for rearing red palm weevils on artificial diet.

No.	Ingredients	Quantity
1	Wheat flour	45 g/L
2	Corn flour	45 g/L
3	Yeast	45 g/L
4	Sorbic acid (Sigma Aldrich, London, UK)	1.6 g/L
5	l-Ascorbic acid (Sigma Aldrich, London, UK)	4 g/L
6	Pharmaton (SITCO Pharma, Riyad, KSA)	2 Capsules/L
7	Tetracycline	500 mg/L
8	Agar (Sigma Aldrich, London, UK)	17.5 g/L
9	Distilled water	1 L

**Table 6:** Primers used for quantitative real-time PCR.

Target Gene	Accession No.	Amplicon Size	Functional Categories	Forward Primer (5′–3′)	Reverse Primer (5′–3′)
*Beta-glucosidase*	KT223628	105 bp	Pathogen Recognition Receptor	TATGGCATGGGCCTTGACTG	GGTGTTCTCGGTCTCTCTGG
*C-type lectin (CTL)*	KT223638	81 bp	Pathogen Recognition Receptor	TGGTACTCCACGCCATCAAC	ATCAGCTACCCACTTTCCGC
*endo-beta-1,4-glucanase*	KT223630	110 bp	Pathogen Recognition Receptor	AGTGACACCTTGGCTTACGG	TTGCCGTTGAGAGCGTTTTG
*Serine protease-like protein*	KT223631	76 bp	Signal Modulation	TTTGTCTGACCGCACCAAGT	TACCGAGCACCATCCACAAC
*Trypsin-like serine protease*	KT223633	113 bp	Signal Modulation	ACAGCTCGGACCAACATGAG	GAAAGAGCTGGGAAGGGTCC
*Calmodulin-like Protein*	KT223632	113 bp	Signal Transduction	GTATCACCACCACCGAGCAA	AACCATGAACTTAGCGGCGA
*EF-hand domain containing protein*	KT223636	88 bp	Signal Transduction	CCAACTGATGGACCACGACA	CTCGTTGGCGATCTTACCGA
*Defensin-like protein*	KT223639	86 bp	Effector	AGGCTGCAGCTATCAAGGAA	AGTGGTGCCTCCATTGTGAC
*Serine carboxypeptidase*	KT223634	108 bp	Effector	CCGAGGAGTACAAAACGGCT	CAGCGTTCCGAACCAGTAGT
*Cathepsin* L.	KT223635	82 bp	Effector	GCCCCTACTCCTTGAACCAC	CCACCCCAGGAGTTCTTGAC
*C-type lysozyme*	KT223629	117 bp	Effector	TAGCACACCAGGCAAAGGTT	TTCGTTGATCCCTTGGCAGT
*Thaumatin-like protein*	KT223637	70 bp	Effector	TCGGAGATGTGGTAGCTTGC	TCCACTACAGCCAGAGGACA
*beta-Actin*	KM438516	129 bp	House-keeping gene	AAAGGTTCCGTTGCCCTGAA	TGGCGTACAAGTCCTTCCTG

## References

[1] Hussain A., Rizwan-ul-Haq M., Al-Jabr A.M., Al-Ayied H.Y. (2013). Managing invasive populations of red palm weevil: A worldwide perspective. J. Food Agric. Environ..

[2] Rasool K., Khan M., Aldawood A., Tufail M., Mukhtar M., Takeda M. (2015). Identification of proteins modulated in the date palm stem infested with red palm weevil (*Rhynchophorus ferrugineus* Oliv.) using two dimensional differential gel spectrometry. Int. J. Mol. Sci..

[3] Francesca N., Alfonzo A., Verde G.L., Settanni L., Sinacori M., Lucido P., Moschetti G. (2015). Biological activity of *Bacillus* spp. evaluated on eggs and larvae of red palm weevil *Rhynchophorus ferrugineus*. Ann. Microbiol..

[4] Güerri-Agulló B., López-Follana R., Asensio L., Barranco P., Lopez-Llorca L.V. (2011). Use of a solid formulation of *Beauveria bassiana* for biocontrol of the red palm weevil (*Rhynchophorus ferrugineus*) (Coleoptera: Dryophthoridae) under field conditions in SE Spain. Fla. Entomol..

[5] Hussain A., Rizwan-ul-Haq M., Al-Ayedh H., Ahmed S., Al-Jabr A.M. (2015). Effect of *Beauveria bassiana* infection on the feeding performance and antioxidant defence of red palm weevil, *Rhynchophorus ferrugineus*. BioControl.

[6] Lo Verde G., Torta L., Mondello V., Caldarella C.G., Burruano S., Caleca V. (2015). Pathogenicity bioassays of isolates of *Beauveria bassiana* on *Rhynchophorus ferrugineus*. Pest Manag. Sci..

[7] Manachini B., Schillaci D., Arizza V. (2013). Biological responses of *Rhynchophorus ferrugineus* (Coleoptera: Curculionidae) to *Steinernema carpocapsae* (Nematoda: Steinernematidae). J. Econ. Entomol..

[8] Moura J., Toma R., Sgrillo R., Delabie J. (2006). Natural efficiency of parasitism by *Billaea rhynchophorae* (Blanchard) (Diptera: Tachinidae) for the control of *Rhynchophorus palmarum* (L.) (Coleoptera: Curculionidae). Neotrop. Entomol..

[9] Hussain A., Tian M.Y., He Y.R., Ahmed S. (2009). Entomopathogenic fungi disturbed the larval growth and feeding performance of *Ocinara varians* (Lepidoptera: Bombycidae) larvae. Insect Sci..

[10] Wang C., St Leger R.J. (2007). The MAD1 adhesin of *Metarhizium anisopliae* links adhesion with blastospore production and virulence to insects, and the MAD2 adhesin enables attachment to plants. Eukaryot. Cell.

[11] Hergannan J.A., Rechhart J.V. (1997). Drosophila immunity. Trends Cell Biol..

[12] Gillespie J.P., Bateman R., Charnley A.K. (1998). Role of cuticle-degrading proteases in the virulence of *Metarhizium* spp. for the desert locust, *Schistocerca gregaria*. J. Invertebr. Pathol..

[13] Tiago P.V., Fungaro M.H.P., Furlaneto M.C. (2002). Cuticle-degrading proteases from the entomopathogen *Metarhizium flavoviride* and their distribution in secreted and intracellular fractions. Lett. Appl. Microbiol..

[14] Hussain A., Li Y.F., Cheng Y., Liu Y., Chen C.C., Wen S.Y. (2013). Immune-related transcriptome of *Coptotermes formosanus* Shiraki workers: The defense mechanism. PLoS ONE.

[15] Mastore M., Arizza V., Manachini B., Brivio M.F. (2015). Modulation of immune responses of *Rhynchophorus ferrugineus* (Insecta: Coleoptera) induced by the entomopathogenic nematode *Steinernema carpocapsae* (Nematoda: Rhabditida). Insect Sci..

[16] Manachini B., Arizza V., Parrinello D., Parrinello N. (2011). Hemocytes of *Rhynchophorus ferrugineus* (Olivier) (Coleoptera: Curculionidae) and their response to *Saccharomyces cerevisiae* and *Bacillus thuringiensis*. J. Invertebr. Pathol..

[17] Shi Z.H., Lin Y.T., Hou Y.M. (2014). Mother-derived trans-generational immune priming in the red palm weevil, *Rhynchophorus ferrugineus* Olivier (Coleoptera, Dryophthoridae). Bull. Entomol. Res..

[18] Mastore M., Rossetti S.B., Giovannardi S., Scari G., Brivio M.F. (2015). Inducible factors with antimicrobial activity after immune challenge in the haemolymph of red palm weevil (Insecta). Innate Immun..

[19] Hussain A., Rizwan-ul-Haq M., Al-Ayedh H., Al-Jabr A. (2014). Mycoinsecticides: Potential and future perspective. Recent Pat. Food Nutr. Agric..

[20] Sevim A., Donzelli B.G.G., Wu D., Demirbag Z., Gibson D.M., Turgeon B.G. (2012). Hydrophobin genes of the entomopathogenic fungus, *Metarhizium brunneum*, are differentially expressed and corresponding mutants are decreased in virulence. Curr. Genet..

[21] Cho E.M., Kirkland B.H., Holder D.J., Keyhani N.O. (2007). Phage display cDNA cloning and expression analysis of hydrophobins from the entomopathogenic fungus *Beauveria* (Cordyceps) *bassiana*. Microbiology.

[22] Zhang S., Xia Y.X., Kim B., Keyhani N.O. (2011). Two hydrophobins are involved in fungal spore coat rodlet layer assembly and each play distinct roles in surface interactions, development and pathogenesis in the entomopathogenic fungus, *Beauveria bassiana*. Mol. Microbiol..

[23] Hussain A., Ruan L., Tian M., He Y. (2009). Pathogenic effect of *Metarhizium anisopliae* on the larval growth and development of *Ocinara varians* Walker (Lepidoptera: Bombycidae). Pak. Entomol..

[24] Martinez D.S.T., Freire M.D.G.M., Mazzafera P., Araujo-Júnior R.T., Bueno R.D., Macedo M.L.R. (2012). Insecticidal effect of labramin, a lectin-like protein isolated from seeds of the beach apricot tree, *Labramia bojeri*, on the Mediterranean flour moth, *Ephestia kuehniella*. J. Insect Sci..

[25] Hussain A., Tian M.Y., He Y.R., Ruan L., Ahmed S. (2010). In vitro and in vivo culturing impacts on the virulence characteristics of serially passed entomopathogenic fungi. J. Food Agric. Environ..

[26] SAS Institute (2000). SAS User’s Guide: Statistics.

[27] Girardin H., Paris S., Rault J., Bellon-Fontaine M.N., Latgé J.P. (1999). The role of the rodlet structure on the physicochemical properties of *Aspergillus* conidia. Lett. Appl. Microbiol..

[28] Abbott W.S. (1925). A method of computing the effectiveness of an insecticide. J. Econ. Entomol..

[29] Analytical Software (2003). Statistix Statistix.

[30] Livak K.J., Schmittgen T.D. (2001). Analysis of relative gene expression data using real-time quantitative PCR and the 2^−ΔΔ*C*t^ Method. Methods.

[31] Scott I.M., Thaler J.S., Scott J.G. (2010). Response of a generalist herbivore *Trichoplusia ni* to jasmonate-mediated induced defense in tomato. J. Chem. Ecol..

